# Impact of climate change on agricultural production; Issues, challenges, and opportunities in Asia

**DOI:** 10.3389/fpls.2022.925548

**Published:** 2022-10-10

**Authors:** Muhammad Habib-ur-Rahman, Ashfaq Ahmad, Ahsan Raza, Muhammad Usama Hasnain, Hesham F. Alharby, Yahya M. Alzahrani, Atif A. Bamagoos, Khalid Rehman Hakeem, Saeed Ahmad, Wajid Nasim, Shafaqat Ali, Fatma Mansour, Ayman EL Sabagh

**Affiliations:** ^1^Institute of Crop Science and Resource Conservation (INRES), Crop Science Group, University of Bonn, Bonn, Germany; ^2^Department of Agronomy, MNS-University of Agriculture, Multan, Pakistan; ^3^Asian Disaster Preparedness Center, Islamabad, Pakistan; ^4^Department of Agronomy, University of Agriculture Faisalabad, Faisalabad, Pakistan; ^5^Department of Biological Sciences, Faculty of Science, King Abdulaziz University, Jeddah, Saudi Arabia; ^6^Princess Dr. Najla Bint Saud Al-Saud Center for Excellence Research in Biotechnology, King Abdulaziz University, Jeddah, Saudi Arabia; ^7^Department of Public Health, Daffodil International University, Dhaka, Bangladesh; ^8^Institute of Plant Breeding and Biotechnology, MNS-University of Agriculture, Multan, Pakistan; ^9^Department of Agronomy, The Islamia University, Bahwalpur, Pakistan; ^10^Department of Environmental Science and Engineering, Government College University, Faisalabad, Pakistan; ^11^Department of Economics, Business and Economics Faculty, Siirt University, Siirt, Turkey; ^12^Department of Agronomy, Faculty of Agriculture, Kafrelsheikh University, Kafrelsheikh, Egypt; ^13^Department of Field Crops, Faculty of Agriculture, Siirt University, Siirt, Turkey

**Keywords:** climate variability, yield reduction, livestock, elevated temperature, adaptation, climate and crop modeling, decision support system, sustainable production

## Abstract

Agricultural production is under threat due to climate change in food insecure regions, especially in Asian countries. Various climate-driven extremes, i.e., drought, heat waves, erratic and intense rainfall patterns, storms, floods, and emerging insect pests have adversely affected the livelihood of the farmers. Future climatic predictions showed a significant increase in temperature, and erratic rainfall with higher intensity while variability exists in climatic patterns for climate extremes prediction. For mid-century (2040–2069), it is projected that there will be a rise of 2.8°C in maximum temperature and a 2.2°C in minimum temperature in Pakistan. To respond to the adverse effects of climate change scenarios, there is a need to optimize the climate-smart and resilient agricultural practices and technology for sustainable productivity. Therefore, a case study was carried out to quantify climate change effects on rice and wheat crops and to develop adaptation strategies for the rice-wheat cropping system during the mid-century (2040–2069) as these two crops have significant contributions to food production. For the quantification of adverse impacts of climate change in farmer fields, a multidisciplinary approach consisted of five climate models (GCMs), two crop models (DSSAT and APSIM) and an economic model [Trade-off Analysis, Minimum Data Model Approach (TOAMD)] was used in this case study. DSSAT predicted that there would be a yield reduction of 15.2% in rice and 14.1% in wheat and APSIM showed that there would be a yield reduction of 17.2% in rice and 12% in wheat. Adaptation technology, by modification in crop management like sowing time and density, nitrogen, and irrigation application have the potential to enhance the overall productivity and profitability of the rice-wheat cropping system under climate change scenarios. Moreover, this paper reviews current literature regarding adverse climate change impacts on agricultural productivity, associated main issues, challenges, and opportunities for sustainable productivity of agriculture to ensure food security in Asia. Flowing opportunities such as altering sowing time and planting density of crops, crop rotation with legumes, agroforestry, mixed livestock systems, climate resilient plants, livestock and fish breeds, farming of monogastric livestock, early warning systems and decision support systems, carbon sequestration, climate, water, energy, and soil smart technologies, and promotion of biodiversity have the potential to reduce the negative effects of climate change.

## Introduction

**Figure 1:**
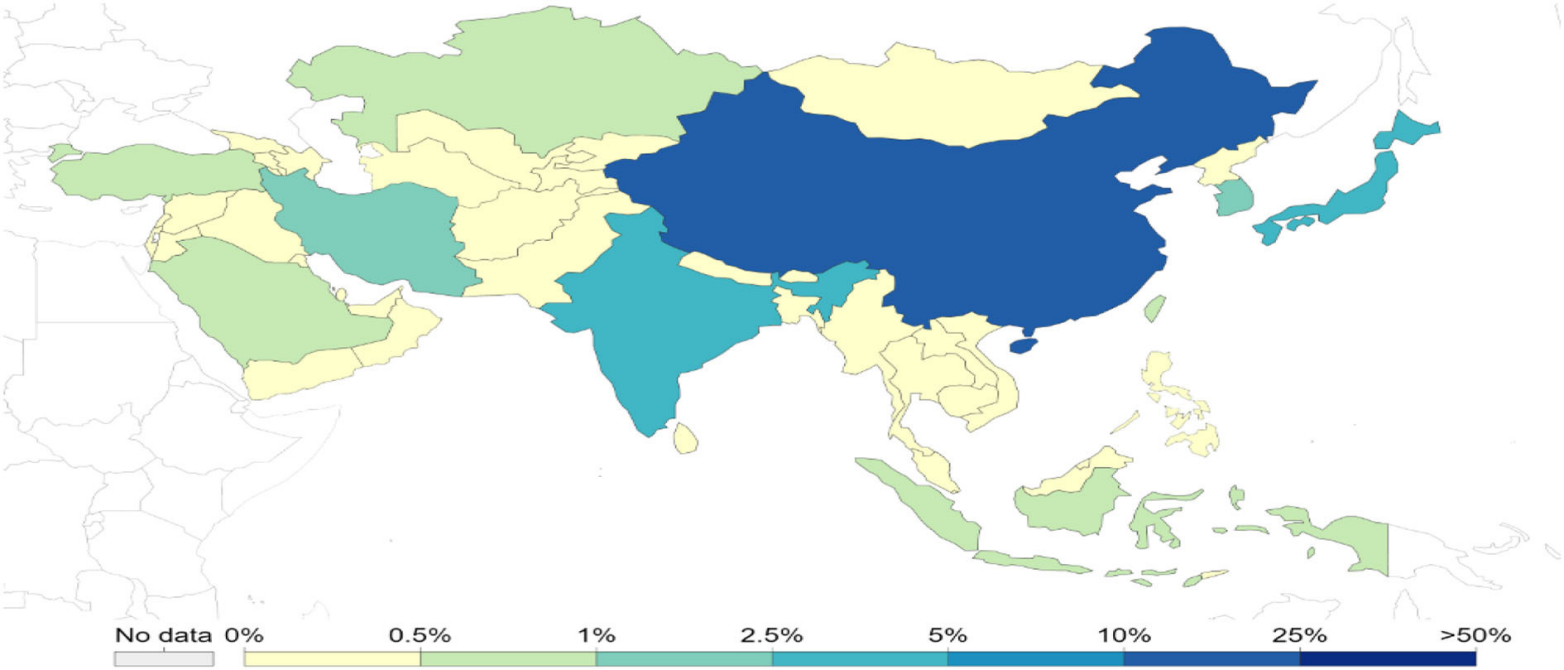
Share of each Asian country in cumulative global carbon dioxide emission (1751–2019; Source: OWID based on CDIAC and Global Carbon Project).

**Figure 2:**
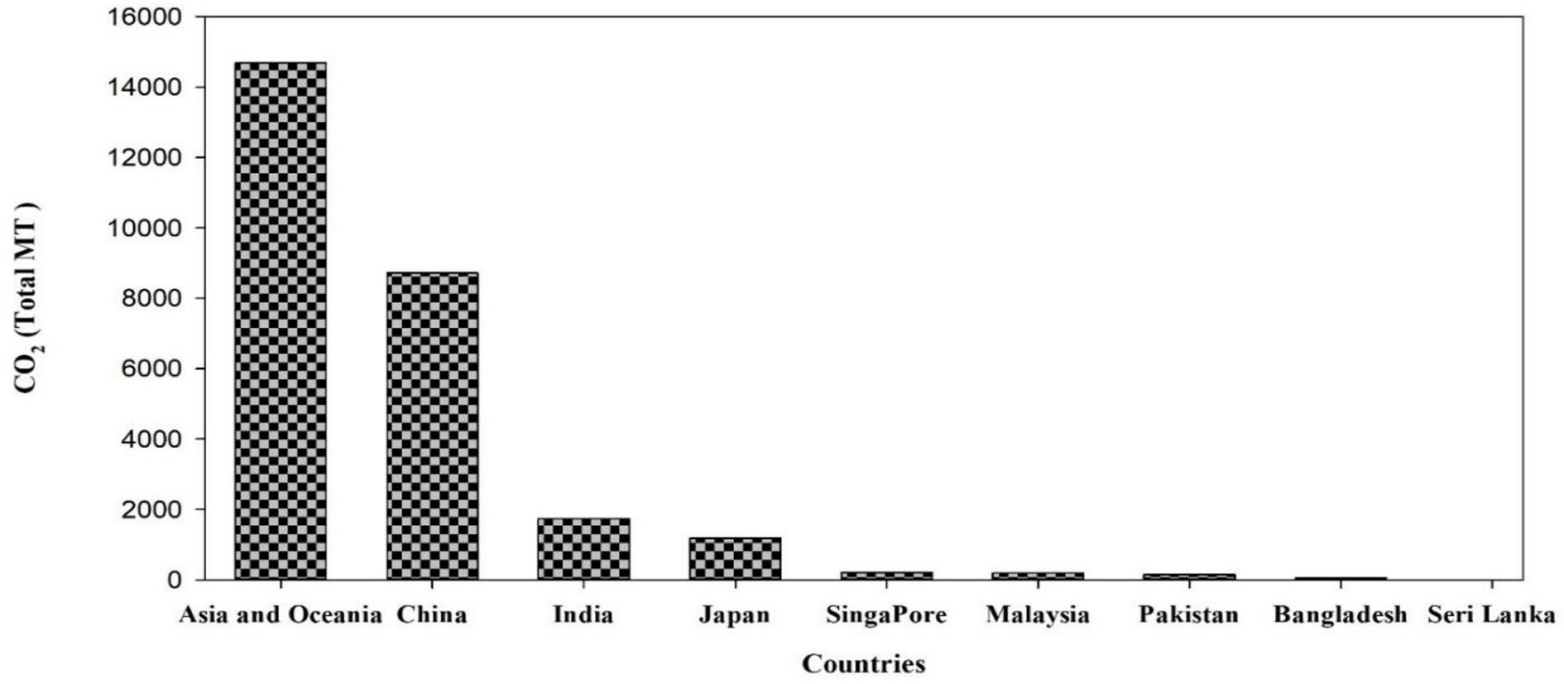
Carbon dioxide (CO_2_) emission from different Asian countries (source: International Energy Statistics https://cdiac.ess-dive.lbl.gov/home.html; Carbon Dioxide Information Analysis Center, Environmental Sciences Division, Oak Ridge National Laboratory, Tennessee, United States).

**Figure 3:**
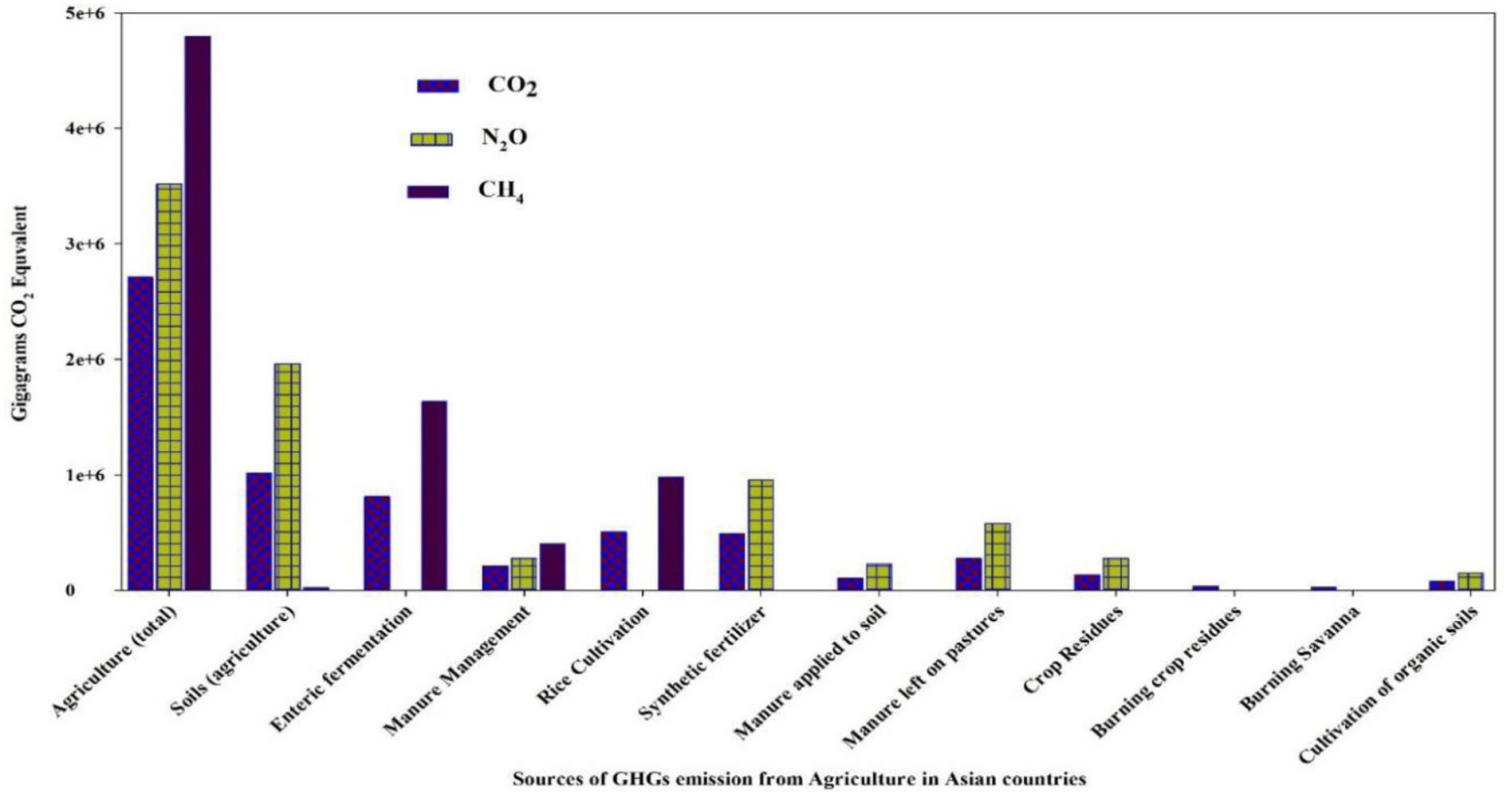
Sources of greenhouse gasses (GHGs) emission from different Asian countries with respect to agricultural components (Source: CAIT climate data explorer *via*. Climate Watch (https://www.climatewatchdata.org/data-explorer/historical-emissions).

**Figure 4:**
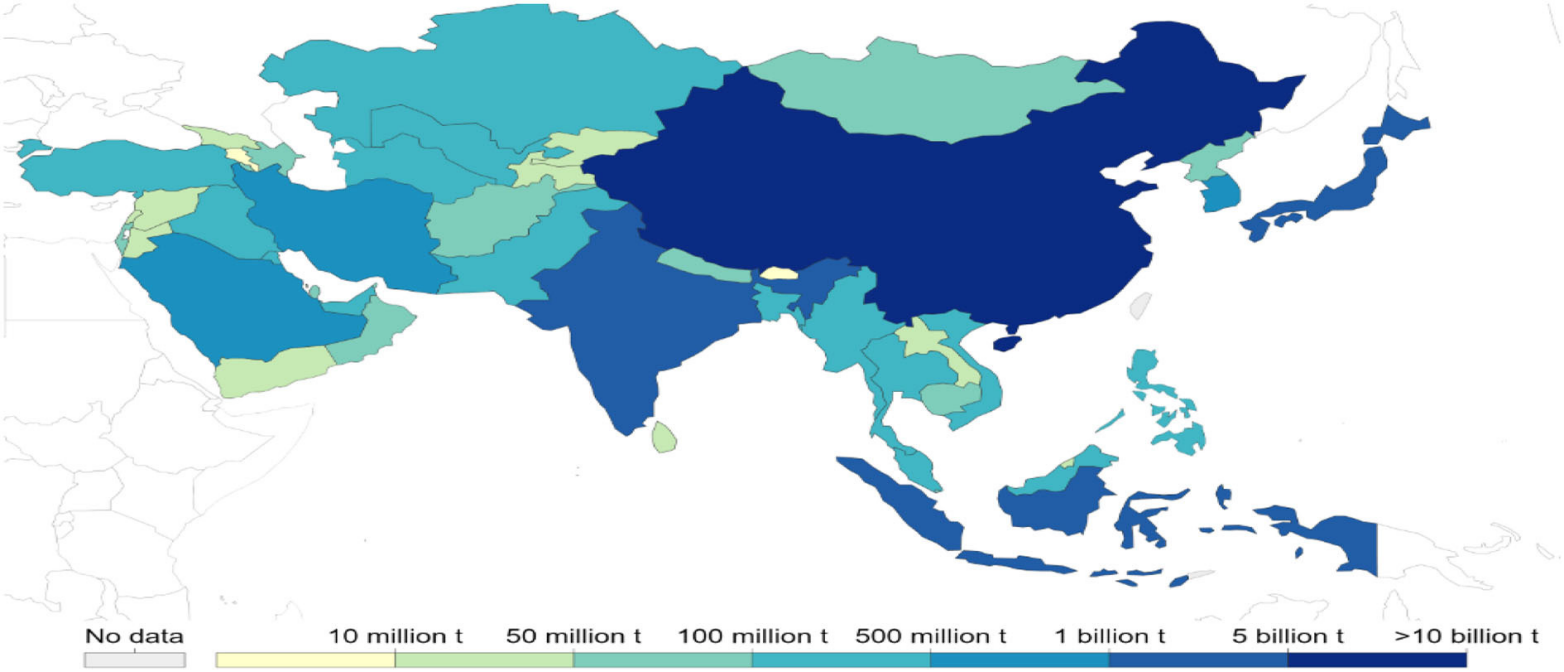
Total greenhouse gasses (GHGs) emission includes emissions from land use changes and forestry from Asian countries (measured in tons of carbon dioxide equivalents [CO_2_-e] (Source: CAIT climate data explorer *via* Climate Watch).

Asia is the most populous subcontinent in the world (UNO, 2015), comprising 4.5 billion people—about 60% of the total world population. Almost 70% of the total population lives in rural areas and 75% of the rural population are poor and most at risk due to climate change, particularly in arid and semi-arid regions (Yadav and Lal, 2018; Population of Asia, 2019). The population in Asia is projected to reach up to 5.2 billion by 2050, and it is, therefore, challenging to meet the food demands and ensure food security in Asia (Rao et al., 2019). In this context, Asia is the region most likely to attribute to population growth rate, and more prone to higher temperatures, drought, flooding, and rising sea level (Guo et al., 2018; Hasnat et al., 2019). In Asia, diversification in income of small and poor farmers and increasing urbanization is shocking for agricultural productivity. Asia is the home of a third of the world's population and the majority of poor families, most of which are engaged in agriculture (World Bank, 2018). We can expect diversification of adverse climate change effects on the agriculture sector due to diversity of farming and cropping systems with dependence on climate. According to the sixth assessment report of IPCC, higher risks of flood and drought make Asian agricultural productivity highly susceptible to changing climate (IPCC, 2019). Climate change has already adversely affected economic growth and development in Asia, although there is low emission of greenhouse gasses (GHG) in this region (Gouldson et al., 2016; Ahmed et al., 2019a). Still, China and India are major contributors to global carbon dioxide emission; the share of each Asian country in cumulative global carbon dioxide emission is presented in Figures 1, 2. Although GHGs emission from the agriculture sector is lower than the others, it still has a negative impact. Emission of GHGs from different agricultural components and contribution to emissions can be found in Figure 3. However, the contribution of Asian countries in GHGs including land use changes and forestry is described in Figure 4.

Asia is facing alarming challenges due to climate change and variability as illustrated by various climatic models predicting the global mean temperature will increase by 1.5°C between 2030 and 2050 if it continues to increase at the current rate (IPCC, 2019). In arid areas of the western part of China, Pakistan, and India, it is also projected that there will be a significant increase in temperature (IPCC, 2019). During monsoon season, there would be an increase in erratic rainfall of high intensity across the region. In South and Southeast Asia, there would be an increase in aridity due to a reduction in winter rainfall. Due to climatic abnormalities, there will be a 0.1 m increase in sea level by 2,100 across the globe (IPCC, 2019). In Asia, an increase in heat waves, hot and dry days, and erratic and unsure rainfall patterns is projected, while dust storms and tropical cyclones are predicted to be worse in the future (Gouldson et al., 2016). Natural disasters are the main reason behind the agricultural productivity (crops and livestock) losses in Asia, including extreme temperature, storms and wildfires (23%), floods (37%), drought (19%), and pest and animal diseases infestation (9%) which accounted for 10 USD billions in amount (FAO, 2015). During the last few decades, tropical cyclones in the Pacific have occurred with increased frequency and intensity. South Asia consisted of 262 million malnourished inhabitants, which made South Asia the most food insecure region across the globe (FAO, 2015; Rasul et al., 2019). In remote dry lands and deserts, the rural population is more vulnerable to climate change due to the scarcity of natural resources.

In Asia, climate variability (temperature and rainfall) and climate-driven extremes (flood, drought, heat stress, cold waves, and storms) have several negative impacts on the agriculture sector (FAO, 2016), especially in the cropping system which has a major role in food security, and thus created the food security issues and challenges in Asia (Cai et al., 2016; Aryal et al., 2019). The rice-wheat cropping system, a major cropping system which fills half of the food demand in Asia, is under threat due to climate change (Ghaffar et al., 2022). Climate change adversely affects both the quantity and quality of wheat and rice crops (Din et al., 2022; Wasaya et al., 2022). For instance, the protein content and grain yield of wheat have been reduced because of the negative impacts of increasing temperature (Asseng et al., 2019). The temperature rise has decreased the crop-growing period, and crop evapotranspiration ultimately reduced wheat yield (Azad et al., 2018). Adverse impacts of climate change and variability on winter wheat yield in China are attributed to increased average temperature during the growing period (Geng et al., 2019). Climate change is also adversely affecting the quality traits especially protein content, and sugars and starch percentages in grains of wheat. Elevated carbon dioxide and high temperatures increase the growth traits while decreasing the protein content in wheat grains (Asseng et al., 2019). Similarly, drought stress also reduces the protein content and soluble sugars of the wheat crop (Rakszegi et al., 2019; Hussein et al., 2022). The decline in the starch content in wheat grains has also been observed under drought stress (Noori and Taliman, 2022). Similarly, heat stress also causes a decline in the protein content, soluble sugar, and starch content in wheat grains (Zahra et al., 2021; Iqbal et al., 2022; Zhao et al., 2022). Climate change also negatively affects the quality of wheat products as the rise in temperature causes a reduction in protein content, sugars, and starch. It is assessed that rise in temperature by 1–4°C could decrease the wheat yield up to 17.6% in the Egyptian North Nile Delta (Kheir et al., 2019). In China, crop phenology has changed because of both climate variability and crop management practices (Liu et al., 2018). Both climate change scenarios and human management practices have adversely affected wheat phenology in India and China (Lv et al., 2013; Ren et al., 2019). The elevated temperature has increased the infestation of the aphid population on wheat crops and ultimately reduced yield (Tian et al., 2019). There is a direct and strong correlation between diseases attached to climate change. For instance, the Fusarium head blight of wheat crops is caused by the Fusarium species and its chances of an attack were increased due to high humidity and hot environment (Shah et al., 2018). A similar study has shown a direct interaction between insect pests and diseases and higher temperature and carbon dioxide levels in rice production (Iannella et al., 2021; Tan et al., 2021; Tonnang et al., 2022).

Climate variability has marked several detriments to rice production in Asia. Climate variability has induced flood and drought, which have decreased the rice yield in South Asia and several other parts of Asia (Mottaleb et al., 2017). Heat stress, drought, flood, and cyclones have reduced the rice yield in South Asia (Cai et al., 2016; Quyen et al., 2018; Tariq et al., 2018). Thus, climate change-driven extremes, particularly heat and drought stress, have also become a serious threat for sustainable rice production globally (Xu et al., 2021). Higher temperatures for a longer period as well as water shortages reduce seed germination which lead to poor stand establishment and seedling vigor (Fahad et al., 2017; Liu et al., 2019). It has been reported that the exposure of rice crops to high temperatures (38°C day/30°C night) at the grain filling stage led to a reduction in grain weight of rice (Shi et al., 2017). Moreover, heat stress also reduces the panicle and spikelet's initiation and ultimately the number of spikelets and grains in the rice production system (Xu et al., 2020). Drought stress also adversely affects the reproductive stages and reduces the yield components especially spikelets per panicle, grain size, and grain weight of rice (Raman et al., 2012; Kumar et al., 2020; Sohag et al., 2020). GLAM-Rice model has projected rice yield will decrease ~45% in the 2080's under RCP 8.5 as compared to 1991–2000 in Southeast Asia (Chun et al., 2016). On the other hand, climate variability could reduce crop water productivity by 32% under RCP 4.5, or 29% under RCP 8.5 by 2080's in rice crops (Boonwichai et al., 2019). In China and Pakistan, high temperature adversely affects the booting and anthesis growth stages of rice ultimately resulting in yield reduction (Zafar et al., 2018; Nasir et al., 2020). Crop models like DSSAT and APSIM have projected a yield reduction of both rice and wheat crops up to 19 and 12% respectively by 2069 due to a rise of 2.8°C in maximum and 2.2°C in minimum temperature in Pakistan (Ahmad et al., 2019).

About 35 million farmers having 3% landholding are projected to convert their source of income (combined crop-livestock production systems) to simply livestock because of the negative impacts of climate change on the quality and quantity of pastures as predicted by future scenarios for 2050 in Asia (Thornton and Herrero, 2010). The livestock production sector also contributes 14.5% of global greenhouse emissions and drives climate variability (Downing et al., 2017). Directly, there would be higher disease infestation and reduced milk production and fertility rates in livestock because of climate extremes like heat waves (Das, 2018; Kumar et al., 2018). Indirectly, heat stress will reduce both the quantity and quality of available forage for livestock. Several studies have reported that heat stress reduces the protein and starch content in the grains of maize which is a widely used forage crop (Yang et al., 2018; Bheemanahalli et al., 2022). Similarly, heat stress also reduces the soluble sugar and protein content in the heat-sensitive cultivars of alfalfa which is also a major forage crop (Wassie et al., 2019). In this context, heat stress leads to a reduction in the quality of forage. There would be an increase in demand for livestock products, however, there would be a decrease in livestock heads under future climate scenarios (Downing et al., 2017). In Asia, a severe shortage of feed for livestock has imposed horrible effects on the livestock population which has been attributed as the result of extreme rainfall variability and drought conditions (Ma et al., 2018).

Timber forests have several significances in Asia, and non-timber forests are also significant sources of food, fiber, and medicines (Chitale et al., 2018). Unfortunately, climate change has imposed several negative impacts on forests at various levels in the form of productive traits, depletion of soil resources, carbon dynamics, and vegetation shifting in Asian countries. In India, forests are providing various services in terms of meeting the food demand of 300 million people, the energy demand of people living in rural areas up to 40%, and shelter to one-third of animals (Jhariya et al., 2019). In Bangladesh, forests are also vulnerable to climate variability as they are facing the increased risks of fires, rise in sea level, storm surges, coastal erosion, and landslides (Chow et al., 2019). Increased extreme drought events with higher frequency, intensity, and duration, and human activities, i.e., afforestation and deforestation, have adversely altered the forest structure (Xu et al., 2018). Hence, there is a need to evaluate climate adaptation strategies to restore forests in Asian countries in order to meet increased demands of food, fiber, and medicines. Agroforestry production is also under threat because of adverse climate change impacts such as depletion of natural resources, predominance of insect pests, diseases and unwanted species, increased damage on agriculture and forests, and enhanced food insecurity (De Zoysa and Inoue, 2014; Lima et al., 2022).

Asia also consists of good quality aquaculture (80% of aquaculture production worldwide) and fisheries (52% of wild caught fish worldwide) which are 77% of the total value addition (Nguyen, 2015; Suryadi, 2020). In Asia, various climatic extremes such as erratic rainfall, drought, floods, heat stress, salinity, cyclone, ocean acidification, and increased sea level have negatively affected aquaculture (Ahmad et al., 2019). For instance, Hilsailisha constituted the largest fishery in Bangladesh, India, and West Bengal and S. Yangi in China have lost their habitat because of climate variability (Jahan et al., 2017; Wang et al., 2019a). Ocean acidification and warming of 1.5°C was closely associated with anthropogenic absorption of CO_2_. Increasing levels of ocean acidity is the main threat to algae and fish. Among various climate driven extremes like drought, flood, and temperature rising, drought is more dangerous as there is not sufficient rainfall especially for aquaculture (Adhikari et al., 2018). Similarly, erratic rainfall, irregular rainfall, storms, and temperature variability have posed late maturity in fish for breeding and other various problems (Islam and Haq, 2018).

The above-mentioned facts have indicated that agriculture, livestock, forestry, fishery, and aquaculture are under threat in the future and can drastically affect food security in Asia. This paper reviews the climate change and variability impacts on the cropping system (rice and wheat), livestock, forestry, fishery, and aquaculture and their issues, challenges, and opportunities. The objectives of the study are to: (i) Review the climate variability impacts on agriculture, livestock, forestry, fishery, and aquaculture in Asia; (ii) summarize the opportunities (adaptation and mitigation strategies) to minimize the drastic effects of climate variability in Asia; and (iii) evaluate the impact of climate change on rice-wheat farmer fields—A case study of Pakistan.

### Impact of climate change and variability on agricultural productivity

#### Impact of climate change and variability on rice-wheat crops

In many parts of Asia, a significant reduction in crop productivity is associated with a reduction in timely water and rainfall availability, and erratic and intense rainfall patterns during the last decades (Hussain et al., 2018; Aryal et al., 2019). Despite the increased crop production owing to the green revolution, there is a big challenge to sustain production and improve food security for poor rural populations in Asia under climate change scenarios (FAO, 2015; Ahmad et al., 2019). In the least developed countries, damage because of climactic changes may threaten food security and national economic productivity (Myers et al., 2017). Yield reductions in different crops (rice, wheat) varied within regions due to variations in climate patterns (Yu et al., 2018). CO_2_ fertilization can increase crop productivity and balance the drastic effects of higher temperature in C_3_ plants (Obermeier et al., 2017) but cannot reduce the effect of elevated temperature (Arunrat et al., 2018). Crop growth and development have been negatively influenced because of rising temperatures and rainfall variability (Rezaei et al., 2018; Asseng et al., 2019).

Rice and wheat are major contributors to food security in Asia. There is a big challenge to increase wheat production by 60% by 2050 to meet ever-enhancing food demands (Rezaei et al., 2018). In arid to semi-arid regions, declined crop productivity is attributed to an increase in temperature at lower latitudes. In China, drought and flood have reduced the rice, wheat, and maize yields and it is projected that these issues will affect crop productivity more significantly in the future (Chen et al., 2018). Rice is sensitive to a gradual rise in night temperature causing yield and biomass to reduce by 16–52% if the temperature increase is 2°C above the critical temperature of 24°C (Yang et al., 2017). In Asia, semi-arid to arid regions are under threat and are already facing the problem of drought stress and low productivity. The quality of wheat produce (protein content, sugars, and starch) and grain yield have reduced because of the negative impacts of increasing temperature and erratic rainfall with high intensity (Yang et al., 2017). In the Egyptian North Nile Delta (up to 17.6%), India, and China, the climate variability has decreased wheat yield significantly which is attributed to a rise in temperature, erratic rainfall and increasing insect pest infestation (Arunrat et al., 2018; Shah et al., 2018; Aryal et al., 2019; Kheir et al., 2019). In South Asia, rice yield in rain-fed areas has already decreased and it might reduce by 14% under the RCP 4.5 scenario while 10% under the RCP 8.5 scenario by 2080 (Chun et al., 2016). High temperature and drought have decreased the rice yield because of their adverse impacts on the booting and anthesis stage in Asia, especially in Pakistan and China (Zafar et al., 2018; Ahmad et al., 2019). Similarly, heat stress is a major threat to rice as it decreases the productive tillers, shrinkage of grains, and ultimately grain yield of rice (Wang et al., 2019b). In Asia, climate change would affect upland rice (10 m ha) and rain-fed lowland rice (>13 million hectares). The projected production of rice and wheat crops by 2030 is presented in Table 1.

**Table 1:** Productivity shock due to climate change and variability on rice and wheat crop production by 2030.

**Countries**	**Rice**	**Wheat**
China	−12 to +12	−10 to +14
Philippines	−10 to +4	−10 to +4
Thailand	−10 to +4	−10 to +4
Rest of SE Asia	−10 to +4	−10 to +4
Bangladesh	−10 to +4	−10 to +4
India	−15 to +4	−10 to +4
Pakistan	−15 to +4	−10 to +4
Rest S Asia	−15 to +4	−10 to +4

Source: Gouldson et al. (2016), Asseng et al. (2019), Chow et al. (2019), Degani et al. (2019), Sanz-Cobena et al. (2019), and Suryadi (2020).

Minus sign (-) indicates the decrease in productivity while positive sign (+) indicates increase in productivity.

#### Impact of climate change and variability on livestock

In arid to semi-arid regions, the livestock sector is highly susceptible to increased temperature and reduced precipitation (Downing et al., 2017; Balamurugan et al., 2018). A temperature range of 10–30°C is comfortable for domestic livestock with a 3–5% reduction in animal feed intake with each degree rise in temperature. Similarly, the lower temperature would increase the requirement feed up to 59%. Moreover, drought and heat stress would drastically affect livestock production under climate change scenarios (Habeeb et al., 2018). Climate variability affects the occurrence and transmission of several diseases in livestock. For instance, Rift Valley Fever (RVF) due to an increase in precipitation, and tick-borne diseases (TBDs) due to a rise in temperature, have become epidemics for sheep, goats, cattle, buffalo, and camels (Bett et al., 2019). Different breeds of livestock show different responses to higher temperature and scarcity of water. In India, thermal stress has negative impacts on the reproduction traits of animals and ultimately poor growth and high mortality rates of poultry (Balamurugan et al., 2018; Chen et al., 2021; van Wettere et al., 2021). In dry regions of Asia, extreme variability in rainfall and drought stress would cause severe feed scarcity (Arunrat et al., 2018). It has been revealed that a high concentration of CO_2_ reduces the quality of fodder like the reduction in protein, iron, zinc, and vitamins B1, B2, B5, and B9 (Ebi and Loladze, 2019). Future climate scenarios show that the pastures, grasslands, feedstuff quality and quantity, as well as biodiversity would be highly affected. Livestock productivity under future climate scenarios would affect the sustainability of rangelands, their carrying capacity and ecosystem buffering capacity, and grazing management, as well as the alteration in feed choice and emission of greenhouse gases (Nguyen et al., 2019).

#### Impact of climate change on forest

Climate variability has posed several negative impacts on forests including variations in productive traits, carbon dynamics, and vegetation shift, as well as the exhaustion of soil resources along with drought and heat stress in South Asian countries (Jhariya et al., 2019; Zhu et al., 2021). In Bangladesh, forests are vulnerable to climate variability due to increased risks of fires, rise in sea level, storm surges, coastal erosion and landslides, and ultimately reduction in forest area (Chow et al., 2019). Biodiversity protection, carbon sequestration, food, fiber, improvement in water quality, and medicinal products are considered major facilities provided by forests (Chitale et al., 2018). In contrast, trait-climate relationships and environmental conditions have drastically influenced structure, distribution, and forest ecology (Keenan, 2015). Higher rates of tree mortality and die-off have been induced in forest trees because of high temperature and often-dry events (Allen et al., 2015; Greenwood et al., 2017; Zhu et al., 2021). For instance, trees Sal, pine trees, and Garjan have been threatened by climate-driven continuing forest clearing, habitat alteration, and drought in South Asian countries (Wang et al., 2019). An increase in temperature and CO_2_ fertilization has increased insect pest infestation for forest trees in North China (Bao et al., 2019). As rising temperature, elevated carbon dioxide (CO_2_), and fluctuating precipitating patterns lead to the rapid development of insect pests and ultimately more progeny will attack forest trees (Raza et al., 2015). Hence, there is a need to develop adaptation strategies to restore forests to meet the increasing demand for food, fiber, and medicines in Asia.

#### Impact of climate change on aquaculture and fisheries

There is a vast difference in response to climate change scenarios of aquaculture in comparison to terrestrial agriculture due to greater control levels over the production environment under terrestrial agriculture (Ottaviani et al., 2017; Southgate and Lucas, 2019). Climatic-driven extremes such as drought, flood, cyclones, global warming, ocean acidification, irregular and erratic rainfall, salinity, and sea level rise have negatively affected aquaculture in South Asia (Islam and Haq, 2018; Ahmad et al., 2019). In Asia, various species such as Hilsa and algae have lost their habitats due to ocean acidification and temperature rise (Jahan et al., 2017). Increased water temperature and acidification of terrestrial agriculture have become dangerous for coral reefs and an increase in average temperature by 1°C for four successive weeks can cause bleaching of coral reefs in India and other parts of Asia (Hilmi et al., 2019; Lam et al., 2019). Ocean warming has caused severe damage to China's marine fisheries (Liang et al., 2018). In Pakistan, aquaculture and fisheries have lost their habitat quality, especially fish breeding grounds because of high cyclonic activity, sea level rise, temperature variability, and increased invasion of saline water near Indus Delta (Ali et al., 2019). It is revealed that freshwater and brackish aquaculture is susceptible to the negative effects of climate variability in several countries of Asia (Handisyde et al., 2017). It is also evaluated that extreme climate variability has deep impacts on wetlands and ultimately aquaculture in India (Sarkar and Borah, 2018).

#### Climate variability and change impact assessment

Agriculture has a complex structure and interactions with different components, which will make it uncertain in a future climate that is a serious risk to food security in the region. Consequently, it is essential to assess the negative impacts of climate change on agricultural productivity and develop adaptive strategies to combat climate change. Simulation models such as General Circulation Models (GCMs) and Representative Concentration Pathways (RCPs) are being used worldwide for the quantification of the negative effects of climate change on agriculture and are supporting the generation of future weather data (Rahman et al., 2018). Primary tools are also available that can estimate the negative impacts of changing climate on crop productivity, crucial for both availability and access to food. Crop models have the potential to describe the inside processes of crops by considering the temperature rise and elevated CO_2_ at critical crop growth stages (Challinor et al., 2018). There are no advanced methods and technologies available to see the impact of climate variability and change on the production of livestock and crops other than the modeling approach (Asseng et al., 2014). There are also modeling tools available, and being used across the world, to quantify the impacts of climate change and variability on crops and livestock production (Ewert et al., 2015; Hoogenboom et al., 2015; Rahman et al., 2019). We decided to quantify the impacts of future climate on farmer's livelihood to study the complete agricultural system by adopting the comprehensive methodology of climate, crop, and economic modeling (RAPs) approaches and found the agricultural model inter-comparison and improvement project (AgMIP) as the best approach.

## A case study—Agricultural model inter-comparison and improvement project

### Impact of climate change on the productivity of rice and wheat crops

Department for International Development (DFID) developed the Agricultural Model Inter-comparison and Improvement Project (Rosenzweig et al., 2013) which is an international collaborative effort to deeply investigate the influences of climate variability and change on crops' productivity in different cropping zones/systems across the world and in Pakistan. The mission of AgMIP is to improve the scientific capabilities for assessing the impact of climate variability on the agricultural production system and develop site-specific adaptation strategies to ensure food security at local to global scales. The review discussed above indicated that the agriculture sector is the most vulnerable due to climatic variability and change. Crop production is under threat in Asian countries—predominantly in developing countries. For instance, Pakistan is also highly vulnerable due to its geographical location with arid to semi-arid environmental conditions (Nasi et al., 2018; Ullah et al., 2019; Ghaffar et al., 2022). There would be impacts that are more adverse in arid and semi-arid regions in comparison to humid regions because of climate change and variability (Nasi et al., 2018; Ali et al., 2019). Future climate scenarios have uncertainty and the projected scenario of climate, especially precipitation, did not coincide with the production technology of crops (Rahman et al., 2018). Floods and drought are anticipated more due to variations in rainfall patterns, and dry seasons are expected to get drier in future. Developing regions of the globe are more sensitive to climate variability and change as these regions implement old technologies whereas developed regions can mediate climate-driven extremes through the implementation of modern technologies (Lybbert and Sumner, 2012). The extent of climate change and variability hazards in Pakistan is massive and may be further shocking in the future. Therefore, it is a matter of time to compute climate variability, impacts on crop production, and develop sustainable adaptation strategies to cope with the negative impact of climate change using AgMIP standards and protocols (AgMIP). The main objective is to formulate adaptation strategies to contradict potential climate change effects and support the livelihood of smallholder farmers in the identified area and circulate this particular information to farmers, extension workers, and policy-makers. Sialkot, Sheikhupura, Nankana sahib, Hafizabad, and Gujranwala are considered the hub of the rice-wheat cropping system (Ghaffar et al., 2022), with an area of 1.1 million hectares. The rice-wheat cropping system is a food basket and its sustainable productivity in future climates will ensure food security in the country and generally overall in the region.

## Methodology of the case study

### Field data collection

**Figure 5:**
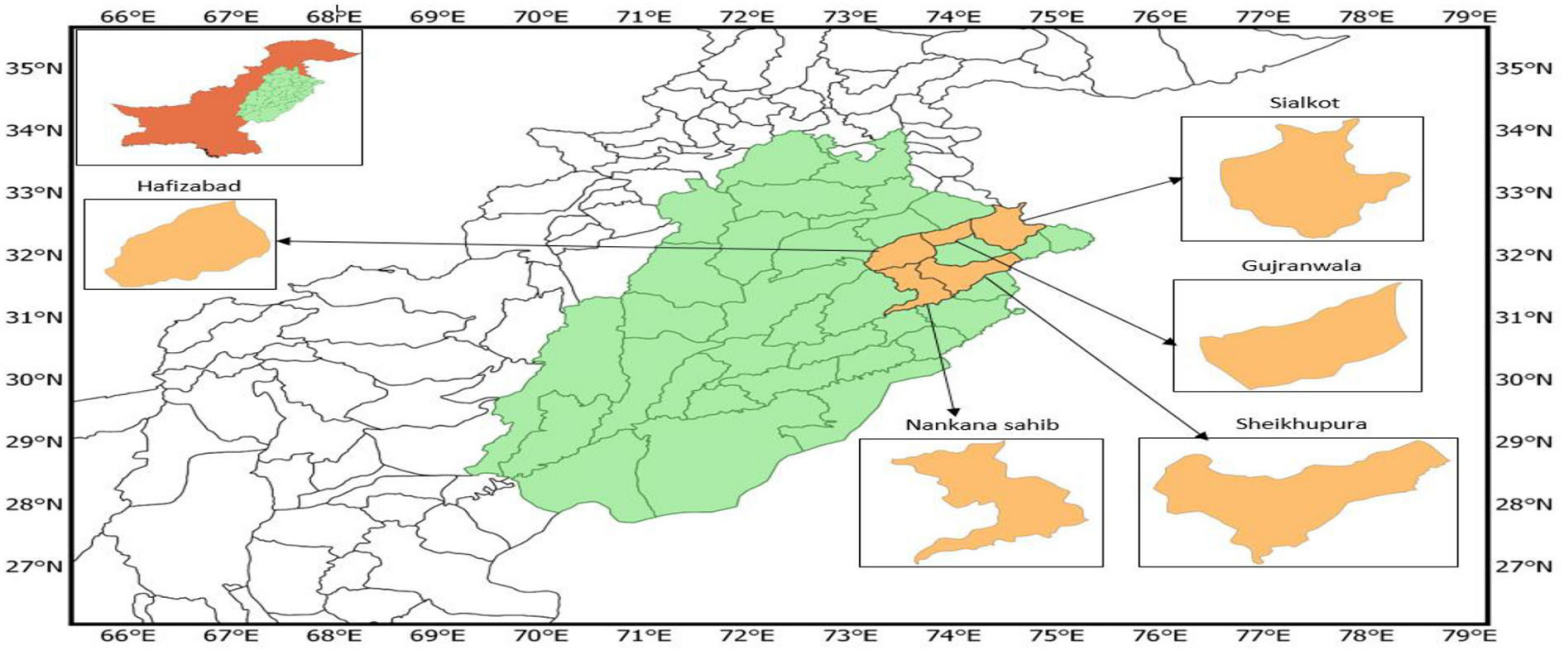
Map of study location/sites in rice-wheat cropping zone of Pakistan.

Field data included the experimental trials and socio-economic data of 155 successive farmers' farms collected during an extensive survey of rice-wheat cropping zone from five-selected districts (Figure 5). From each district, randomly two villages were selected from each division, randomly 30 respondents and 15 farms of true representation of the farming population from each village considered. Crop management data included all agronomic practices from sowing to harvesting such as planting time, planting density, fertilizers amount and organic matter amendment, irrigation amount and intervals, cultural operations, grain yield, and biomass production collected for both crops, rice and wheat, and overall, for all systems. Farm data for the rice-wheat cropping system were analyzed with crop and economic models to see the impact of climate variability on crop production.

### Historic and future climatic data

Daily historic data was collected from the Pakistan Meteorological Department (PMD) for all study locations. The quality of observed weather data was checked following the protocol of the Agricultural Model Inter-comparison and Improvement Project (AgMIP) protocols (AgMIP, 2013). Station-based downscaling was performed with historic weather data from all study sites/locations in the rice-wheat cropping zone. For the zone/region, five GCMs (CCSM4, GFDL-ESM2M, MIROC5, HadGEM2-ES, and MPI-ESM-MR) of the latest CMIP5 family were engaged for the generation of climate projections for the mid-century period using the RCP 8.5 concentration scenario, and using the protocols and methodology developed by AgMIP (Ruane et al., 2013, 2015; Rahman et al., 2018). GCMs were selected on the basis of different factors such as better performance in monsoon seasons, the record of accomplishment of publications, and the status of the model-developing institute. Under the RCP 8.5 scenario, an indication of warming ranges 2–3°C might be expected in all selected districts for the five CMIP5, GCMs in comparison to the baseline between the periods of 2040–2069. However, there is no uniform warming recorded under all 5 CMIP5 GCMs. For instance, CCSM4 and GFDL-ESM-2M showed uniform increased temperatures during April and September months. The outputs of the GCMs indicated large variability in the estimated values of precipitation. The HadGEM2-ES and GFDL-ESM2M projected mean of 200 and 100 mm between times 2040–2069, respectively. On average, a minor rise in annual rainfall (mm) is indicated by five GCMs in comparison to the baseline.

### Crop models (DSSAT and APSIM)

To understand the agronomic practices and the impact of climate variability on the development and growth of plants, crop simulation models like DSSATv4.6 (Hoogenboom et al., 2015, 2019) and APSIMv7.5 (Keating et al., 2003) were applied. Three field trials were conducted on rice and wheat crops during two growing seasons, to collect the data like phenology, crop growth (leaf area index, biomass accumulation), development, yield, and agronomic management data by following the standard procedure and protocols. Crop models are calibrated with experimental field data (phenology, growth, and yield data) under local environmental conditions by using soil and weather data. Crop models were further validated with farmers' field data of rice and wheat crops. Climate variability impact on both crops was assessed with historic data (baseline) and future climate data of mid-century in this region.

### Tradeoff analysis model for multi-dimensional impact assessment

**Figure 6:**
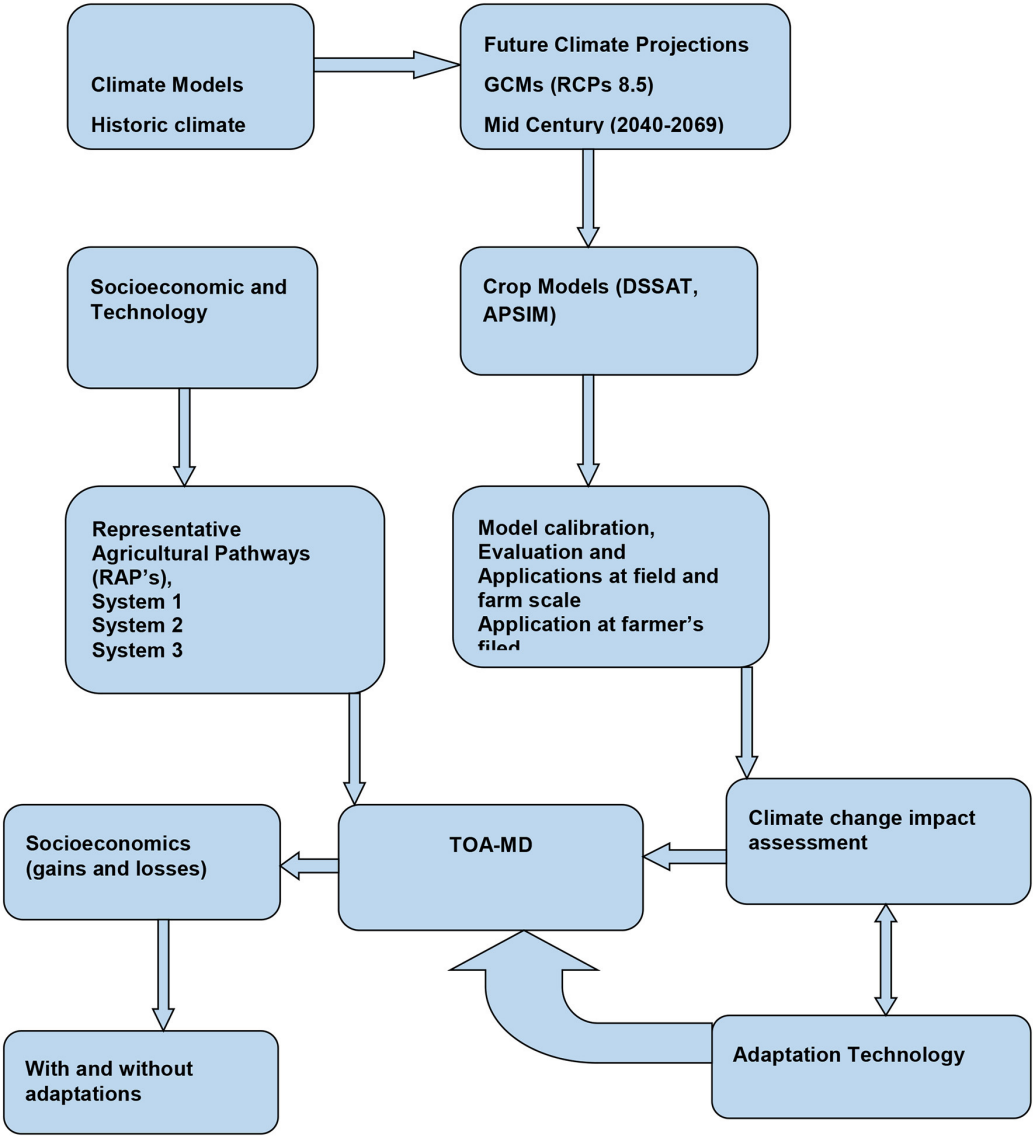
Schematic illustration showing connections between the different models (climate, crop, and economic) and the points of contact between them in terms of input-output and climate analysis.

For the analysis of climate change impact socio-economic indicators, version 6.0.1 of the Tradeoff Analysis Model for Multi-Dimensional Impact Assessment (TOA-MD) Beta was employed (Antle, 2011; Antle et al., 2014). It is an economical and standard model employed for the analysis of technology adoption impact assessment and ecosystem services. Schematically illustrated, showing connections between the different models and the points of contact between them in terms of input-output in a different climate, crop and economic models and climate analysis is shown in Figure 6. Various factors that may affect the anticipated values of the production system are technology, physical environment, social environment, and representative agricultural pathways (RAPs), hence it is necessary to distinguish these factors (Rosenzweig et al., 2013). RAPs are the qualitative storylines that can be translated into model parameters such as farm and household size, practices, policy, and production costs. For climate impact assessment, the dimensionality of the analysis is the main threat in scenario design. Farmers employ different systems for operating a base technology. For instance, system 1 included base climate, in system 2, farmers use hybrid climate, and in system 3, farmers use perturbed climate to cope with future climate with adaptation technology. The analysis gave the answer to three core questions (Rosenzweig et al., 2013). First, without the application RAPs of the core question, one-climate change impact assessments (CC-IA) were formulated. Second, analysis was again executed for examining the negative effects of climate change on future production systems. Third, analysis was executed for future adapted production systems through RAPs and adaptations. Two crop models, i.e., DSSAT and APSIM, outputs were used as the inputs of TOA-MD. Different statistical analyses like root mean square error (RMSE), mean percentage difference (MPD) d-stat, percent difference (PD), and coefficient of determination (R2) were used to check the accuracy of models.

## Results

### Farmers field data validation

Crop model simulation results regarding calibration and validation of both crops (rice and wheat) were in good agreement with the field experimental data. Both models were further validated using farmers' field data of rice and wheat crops in rice-wheat cropping zone after getting robust genetic coefficients. Model validation results of 155 farmers of rice and wheat crops indicated the good accuracy of both models (DSSAT, APSIM) and have a good range of statistical indices. Both of these crop models showed an improved ratio between projected and observed rice yield in farmers' fields with RMSE 409 and 440 kg ha^−1^ and d-stat 0.80 and 0.78, respectively. Similarly, the performance of models DSSAT and APSIM for a yield of wheat was also predicted with RMSE of 436 and 592 kg ha^−1^ and d-stat of 0.87, respectively.

### Quantification of climate change impact by crop models

**Figure 7:**
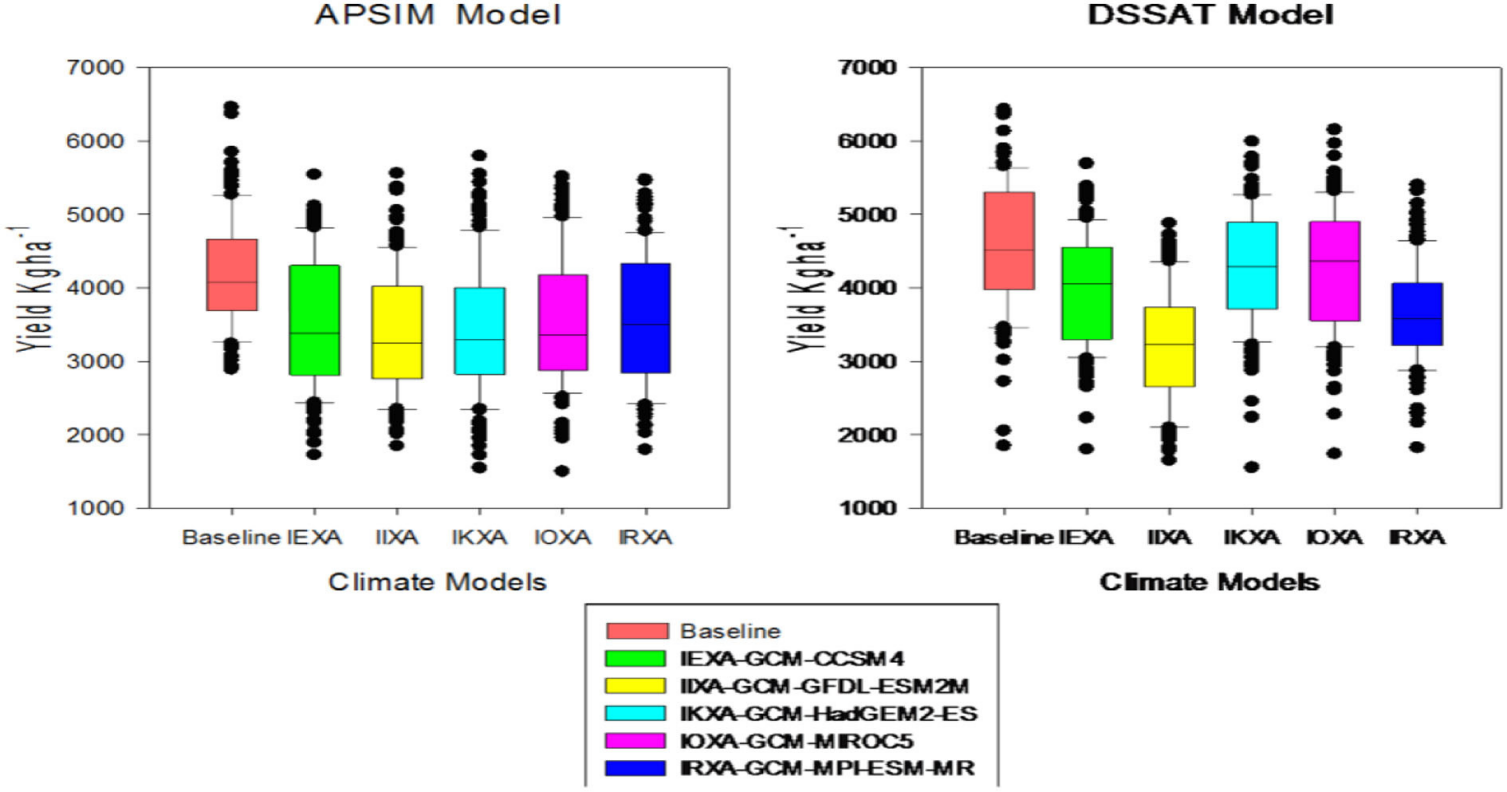
Reduction in rice yield of APSIM and DSSAT models for 155 farms; variation with 5-GCMs in rice-wheat cropping system of Punjab-Pakistan.

**Figure 8:**
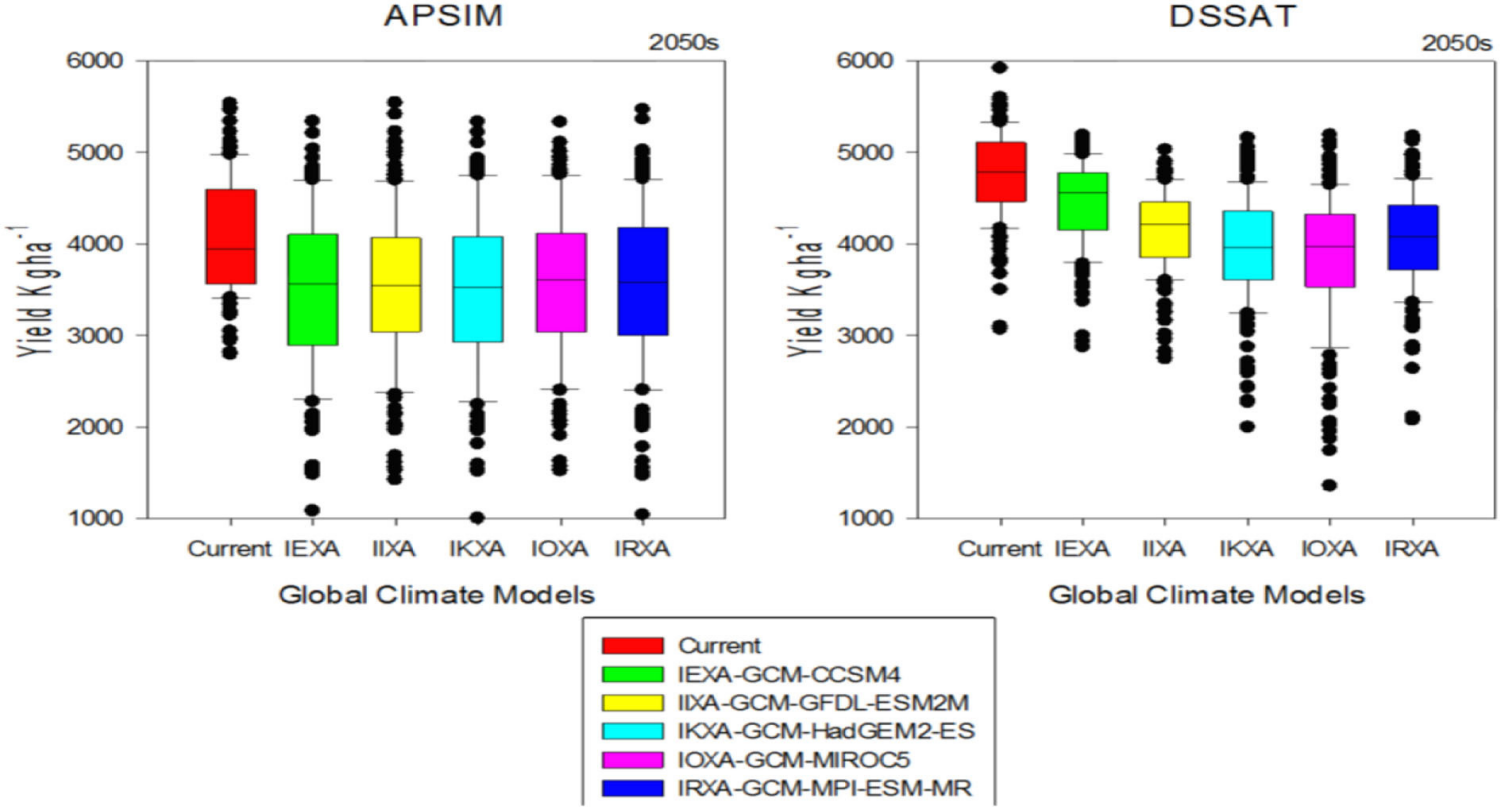
Reduction in wheat yield of APSIM and DSSAT models for 155 farms; variation with 5-GCMs in rice-wheat cropping system of Punjab-Pakistan.

Climate change impact assessment results in the rice-wheat cropping zone of 155 farms indicated that yield reduction varied due to differences in GCM's behavior and variability in climatic patterns. It is predicted that mean rice yield reduction would be up to 15 and 17% for DSSAT and APSIM respectively during mid-century while yield reduction variation among GCMs are presented in Figure 7. Rice indicated a yield decline ranging from 14.5 to 19.3% for the case of APSIM while mean yield reduction of the rice crop was between 8 and 30% with DSSAT. Reduction in production of wheat varied among GCMs as well as an overall reduction in yield in rice-wheat cropping systems. For wheat, with DSSAT would be a 14% reduction whereas for APSIM, the reduction would be 12%. GCMs reduction in wheat yield for midcentury (2040–2069) is shown in Figure 8. Reduction in wheat yield for all 5 GCMs was from 10.6 to 12.3% in the case of APSIM while mean reduction in wheat yield was between 6.2 and 19%. As rice is a summer crop where the temperature is already high and, according to climate change scenarios, there is an increase in both maximum and minimum temperature, an increase in minimum temperature leads to more reduction in yield as compared to wheat being a winter season crop. It was hypothesized that the increase in night temperature (minimum temperature) leading to more losses in the summer season may be due to high temperature, particularly at anthesis and grain formation stages in rice crops, as it is already an irrigated crop and rainfall variability (more rainfall) cannot reduce the effect of high temperature in the rice yield as compared to the wheat crop.

### Climate change economic impact assessment and adaptations

#### Sensitivity of current agricultural production systems to climate change

Climate change is damaging the present vulnerabilities of poor small farmers as their livelihood depends directly on agriculture. Noting various impacts of future climate (2040–2069) on a current production system (current technologies), we examine the vulnerability of the current production system used for the assessment of the adverse impacts of climate change on crop productivity and other socio-economic factors. Climate change impacts possible outcomes for five GCMs based on the estimation of yield generated by two crop models presented in Table 2. In Table 3, and the grain losses and net impacts as a percentage of average net returns for the first core question are given for each GCM. The analysis clearly shows the observed values of the mean yield of wheat and rice, which are estimated to be 18,915 kg and 18,349 kg/ farm respectively in the projected area. For all GCMs, observed average milk production was 3,267 liters per farm with a 12% average decline in yield found under livestock production. Losses were about 69–83% and from 72 to 76% for DSSAT and APSIM respectively as predicted by TOA-MD analysis because of the adverse effects of climate change situations. For DSSAT, percentage losses and gains in average net farm returns were from 13 to 15% and 23 to 30%, respectively. While gains were 14–15% and losses were from 25 to 27%, respectively for APSIM. Without adverse impacts of climate change, a net income of Rs. 0.54 per farm pragmatic was predicted by DSSAT and APSIM. However, DSSAT predicted Rs. 0.42–0.48 M per farm and APSIM predicted Rs. 0.45–0.47 M net income per farm under climate change for all GCMs. An increase in the poverty rate in climate change situations would be 33–38% for DSSAT and it would be 35–37% for APSIM, respectively while the rate of poverty with no adverse impacts of climate change would be 29%.

**Table 2:** Relative yield summary of crop models.

		**Time averaged relative yield (r) of GCM**		
**Crop**	**Crop Model**	**CCSM4**	**GFDL-ESM2M**	**HadGEM2-ES**	**MIROC5**	**MPI-ESM-MR**	**Mean**
Rice	DSSAT	0.90	0.72	0.95	0.87	0.79	0.85
	APSIM	0.83	0.80	0.80	0.83	0.85	0.82
Wheat	DSSAT	0.93	0.83	0.83	0.80	0.85	0.82
	APSIM	0.90	0.90	0.90	0.91	0.91	0.90

r = ∑s2/∑s1, ∑s2, Time averaged mean of simulated future yield; ∑s1, Time averaged mean of simulated past yield.

**Table 3:** Aggregated gains and losses with CCSM4 GCM (without adaptation and with trend) of DSSAT and APSIM.

**Crop model**	**Poverty rate (%) with climate change**	**Losses (%)**	**Gains (% mean net returns)**	**Losses (% mean net returns)**	**Net impacts (% mean net returns)**
DSSAT	16.6	57.0	13.2	15.6	−2.4
APSIM	19.1	63.2	13.4	18.5	−5.1

#### Impacts of climate change on future agricultural production systems

In regard to the second core question, a comparison of system 1 (current climate and future production system) with system 2 (future climate and future production system in mid-century) was analyzed with the aid of TOA-MD using 5 GCMs. Mean wheat and rice yield reduction for DSSAT was from 6.2 to 19% and 8 to 30% respectively, and APSIM indicated a decline ranging from 10.6 to 12.3% and 14 to 19%, respectively. For all analyses of Q2, the projected mean yield was 25,073 kg per farm under rice production. While in the case of livestock for all analyses, the mean projected milk production was 3,267 L/farm with its mean decline in yield estimated to be about 12%. Percentage losses for DSSAT and APSIM would fluctuate between 57 and 70% and from 61 to 71%, respectively for all five GCMs.

Mean net farm returns for gains and losses, as a percentage for DSSAT would be 11–13% and from −16 to −22%, respectively. While the percentage of gains and losses would be between 10 and 15% and −17% and −19% in the case of APSIM, respectively. DSSAT predicted Rs. 89–100 thousand per person while APSIM predicted Rs. 93–97 thousand per person per capita income in changing climatic scenarios. For both crop models, the poverty rate will be 16% without climate change. While poverty rates will be from 17 to 19% in the case of DSSAT and ranging from 18 to 19% for APSIM with climate change (Table 3).

#### Evaluation of potential adaptation strategies and representative agricultural pathways

**Figure 9:**
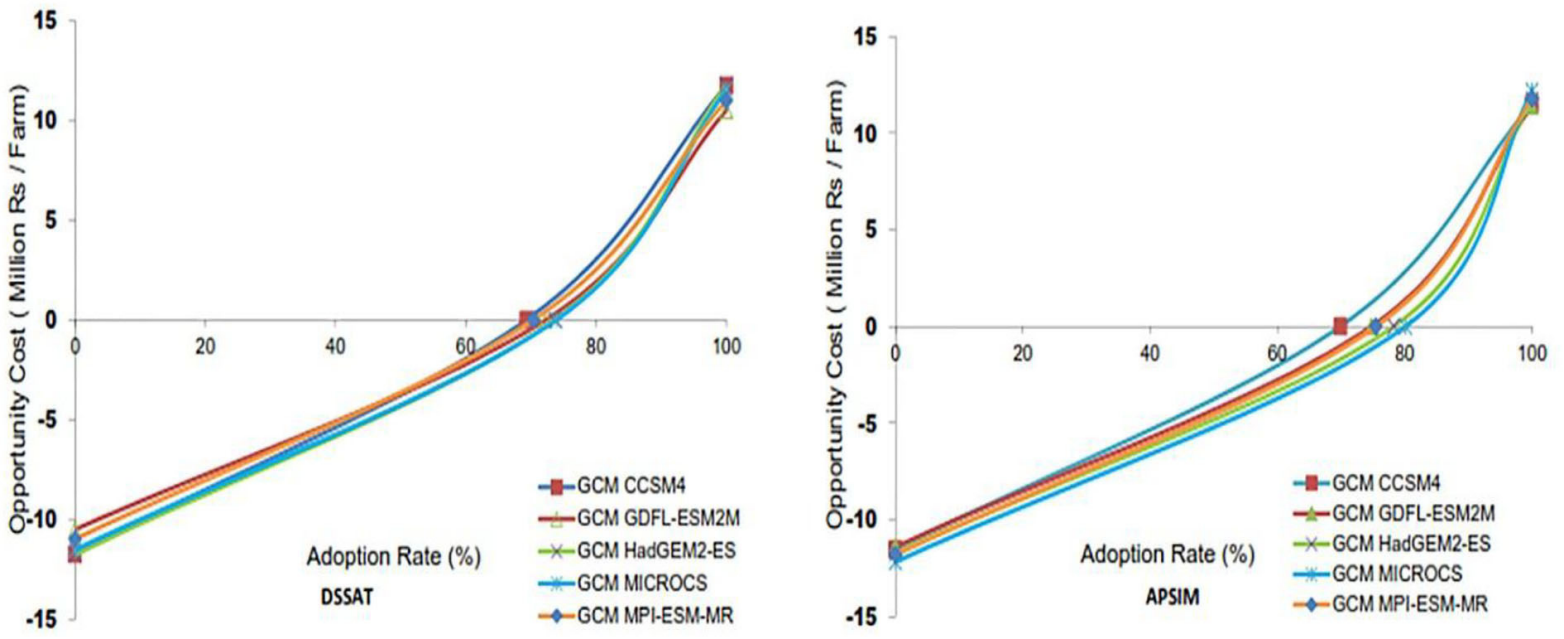
Distribution of adopters and non-adopters for all 5 GCMs (with adaptation and with trend). The percentage of adopters due to adaptation technologies for DSSAT and APSIM in rice-wheat cropping system would be between 92 and 93% and 93 and 94%, respectively. For DSSAT and APSIM estimated per head income with adaptation cases will be from Rs. 89 to 100 and 93 to 97 thousand and from Rs. 156 to 174 and 166 to 181 thousand per head respectively in a year.

Adaptation technologies for rice and wheat crops (Table 4) are used in crop growth models and economic TOA-MD model analysis (Table 5) for simulating the sound effects of prospective adaptation strategies on both adapters and non-adapters distribution. This TOA-MD analysis compared “system 1” (incorporating RAPs) and “system 2” (incorporating RAPs and adapted technology) for the rice-wheat system in the mid-century based on crop models DSSAT and APSIM using 5 GCMs. The mean yield change of wheat and rice crops was from 60 to 72% for DSSAT and 70 to 80% for APSIM respectively, wheat crop indicated a change that ranges from 80 to 89% and 62 to 84% for all five GCMs (Figure 9). Under livestock production, the estimated average production of milk exclusive of adaptation was 3,593 liters/farm for all analyses and for all cases indicates a 42% increase in average yield. The percentage of adopters due to adaptation technologies for DSSAT and APSIM in rice-wheat cropping systems would be between 92 and 93% and 93 and 94%, respectively. For DSSAT and APSIM estimated per head income with adaptation cases will be from Rs. 89 to 100 and 93 to 97 thousand and from Rs. 156 to 174 and 166 to 181 thousand per head, respectively in a year. Without and with adaptation, poverty would range between 17 and 19% and 12 and 13% respectively, for DSSAT and from 18 to 19% and 12 to 13%, respectively for APSIM (Table 6). Climatic changes in the rice-wheat cropping areas of Punjab province will have less impact on the future systems after implementing the adaptation strategies, with a large and significant impact imposed by these adaptations.

**Table 4:** Adaptation technology related to crop management used for crop models (DSSAT and PSIM) to cope with the negative impacts of climate change during mid-century (2040–2069).

**Sr. #**	**Variable**	**Direction of change**	**% change**	
			**Rice**	**Wheat**
1	Nitrogen/hectare (Kg)	Increase	15	25
2	Sowing density (Plant/m2)	Increase	15	30
3	Irrigation	Decrease	15	25
4	Sowing dates	Decrease	5 days	15 days
5	Overall productivity	Increase	55	60

Percentage change (% change) shows the percentage of farmers using the crop management practices related to crop models to reduce the adverse effects of climate change.

**Table 5:** Adaptation technology related to socioeconomic used for crop models (DSSAT and APSIM) to cope with the negative impacts of climate change during mid-century (2040–2069).

**Sr. #**	**Variable**	**Direction of change**	**% change**	
			**Rice**	**Wheat**
1	Average household persons	Increase	40	40
2	Non-agricultural income	Increase	40	40
3	Price of output	Increase	65	70
4	Variable production cost	Increase	55	50

Percentage change (% change) shows the percentage of farmers using the socioeconomic technology related to crop models to reduce the adverse effects of climate change.

**Table 6:** Projected adoption of adaptation package used in crop models for CCSM4 GCM during mid-century.

**Economic indicators**	**DSSAT**		**APSIM**	
	**Projected adoption rate** = **69.3%**		**Projected adoption rate** = **70%**	
	**Without adaptation**	**With adaptation**	**Without adaptation**	**With adaptation**
Mean farm net returns (million Rs./farm/year)	1.1	1.29	1.06	1.29
Per capita income (thousand Rs./person/year)	100	117	95	115
Poverty rate (%)	16.6	16	19.1	16

## Discussion

### Opportunities in the era of climate change for agriculture

#### Scope of adaptation and mitigation strategies for sustainable agricultural production

It is essential to assess the impact of climate variability on agricultural productivity and develop adaptation strategies/technology to cope with the negative effects to ensure sustainable production. The hazardous climate change effects can be reduced by adapting climate-smart and resilient agricultural practices, which will ensure food security and sustainable agricultural production (Zafar et al., 2018; Ahmad et al., 2019; Ahmed et al., 2019b). Adaptation is the best way to handle climate variability and change as it has the potential to minimize hazardous climate change effects for sustainable production (IPCC, 2019). Innovative technologies and defensive adaptation can reduce the uncertain and harmful effects of climate on agricultural productivity.

**Figure 10:**
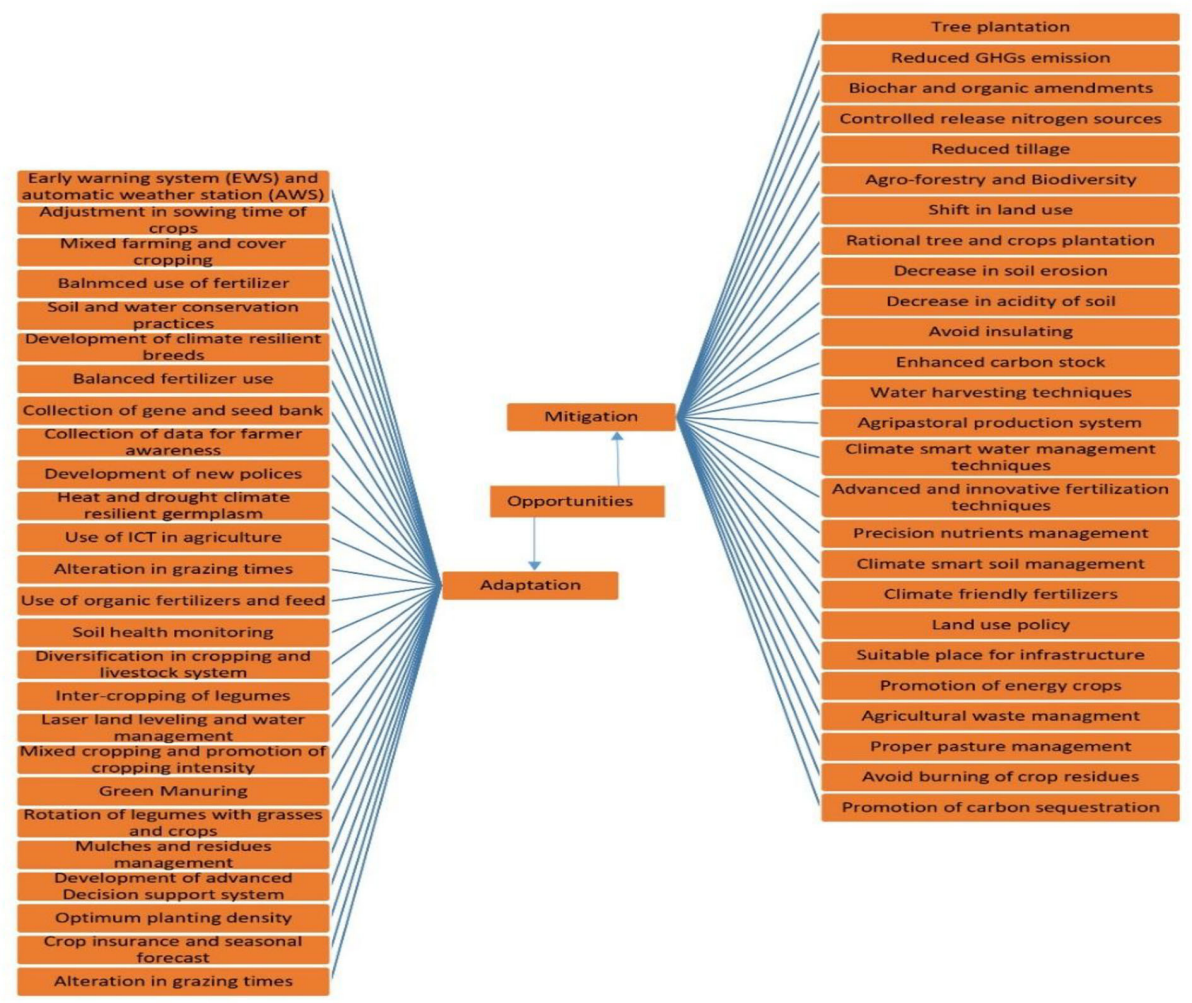
Overview of opportunities including adaptations and mitigations strategies for sustainable agriculture production system in Asia.

Therefore, to survive the harmful climate change effects, the development and implementation of adaptation strategies are crucial. In developing countries, poverty, food insecurity and declined agricultural productivity are common issues, which indicate the need for mitigation and adaptation measures to sustain productivity (Clair and Lynch, 2010; Lybbert and Sumner, 2012; Mbow et al., 2014). At the national and regional level, the insurance of food security is the major criterion for the effectiveness of mitigation and adaptation. Integration of adaptation and mitigation strategies is a great challenge to promote sustainability and productivity. Climate resilient agricultural production systems can be developed and diversified with the integration of land, water, forest biodiversity, livestock, and aquaculture (Hanjra and Qureshi, 2010; Meena et al., 2019). Summary and overview of all below discussed potential opportunities are presented in Figure 10.

### Reduction in GHGs emission

Reduction in GHGs emissions from agriculture under marginal conditions and production of more food are the major challenges for the development of adaptation and mitigation measures (Smith and Olesen, 2010; Garnett, 2011; Fujimori et al., 2021). Similarly, it is an immediate need to control such practices in agriculture which lead to GHGs emissions, i.e., N_2_O emissions from the application of chemical fertilizers, and CH_4_ emissions from livestock and rice production systems (Herrero et al., 2016; Allen et al., 2020). Similarly, alternate wetting and drying and rice intensification are important to reduce the GHGs emission from rice crops (Nasir et al., 2020). Carbon can be restored in soil by minimizing the tillage, reducing soil erosions, managing the acidity of the soil, and implementing crop rotation. By increasing grazing duration and rotational grazing of pastureland, sequestration of carbon can be achieved (Runkle et al., 2018). About 0.15 gigatonnes of CO_2_ equal to the amount of CO_2_ produced in 1 year globally, can be sequestered by adopting appropriate grazing measures (Henderson et al., 2015). Development of climate-resilient breeds of animals and plants with higher growth rates and lower GHGs emissions should be developed to survive under harsh climatic conditions. Focus further on innovative research and development for the development of climate-resilient breeds, especially for livestock (Thornton and Herrero, 2010; Henry et al., 2012; Phand and Pankaj, 2021).

### Application of ICT and decision support system

To mitigate and adapt to the drastic effects of climate variability and change, information and communication technologies (ICTs) can also play a significant role by promoting green technologies and less energy-consuming technology (Zanamwe and Okunoye, 2013; Shafiq et al., 2014; Nizam et al., 2020). Timely provision of information from early warning systems (EWS) and automatic weather stations (AWS) on drought, floods, seasonal variability, and changing rainfall patterns can provide early warning about natural disasters and preventive measures (Meera et al., 2012; Imam et al., 2017), and it can also support farmers' efforts to minimize harmful effects on the ecosystems. Geographical information systems (GIS), wireless sensor networks (WSN), mobile technology (MT), web-based applications, satellite technology and UAV can be used to mitigate and adapt to the adverse effects of climate change (Kalas, 2009; Karanasios, 2011). Application of different climate, crop, and economic models may also help reduce the adverse effects of climate variability and change on crop production (Hoogenboom et al., 2011, 2015, 2019; Ewert et al., 2015).

### Crop management and cropping system adaptations

Adaptation strategies have the potential to minimize the negative effect of climate variability by conserving water through changes in irrigation amount, timely application of irrigation water, and reliable water harvesting and conservation techniques (Zanamwe and Okunoye, 2013; Paricha et al., 2017). Crop-specific management practices like altering the sowing times (Meena et al., 2019), crop rotation, intercropping (Hassen et al., 2017; Moreira et al., 2018), and crop diversification and intensification have a significant positive contribution as adaptation strategies (Hisano et al., 2018; Degani et al., 2019). Meanwhile, replacement of fossil fuels by introducing new energy crops for sustainable production (Ruane et al., 2013) is also crucial for the sustainability of the system. Different kinds of adaptation actions (soil, water, and crop conservation, and well farm management) should be adapted in case of long-term increasing climate change and variability (Williams et al., 2019). Similarly, alteration in input use, changing fertilizer rates for increasing the quantity and quality of the produce, and introduction of drought resistant cultivars are some of the crucial adaptation approaches for sustainable production. Therefore, under uncertain environmental conditions, to ensure sustainable productivity, crops having climatic resilient genetic traits should also be introduced (Bailey-Serres et al., 2018; Raman et al., 2019). Similarly, to ensure the sound livelihood of farmers, it is important to develop resilient crop management as well as risk mitigation strategies.

### Opportunities for a sustainable livestock production system

The integration of crop production, rearing of livestock and combined use of rice fields for both rice and fish production lead to enhancing the farmers' income through diversified farming (Alexander et al., 2018; Poonam et al., 2019). Similarly, variations in pasture rates and their rotation, alteration in grazing times, animal and forage species variation, and combination production of both crops and livestock are the activities related to livestock adaptation strategies (Kurukulasuriya and Rosenthal, 2003; Havlik et al., 2013). Under changing climate scenarios, sustainable production of livestock should coincide with supplementary feeds, management of livestock with a balanced diet, improved waste management methods, and integration with agroforestry (Thornton and Herrero, 2010; Renaudeau et al., 2012).

### Carbon sequestration and soil management

Selection of more drought-resilient genotypes and combined plantation of hardwood and softwood species (Douglas-fir to species) are considered adaptive changes in forest management under future climate change scenarios (Kolstrom et al., 2011; Hashida and Lewis, 2019). Similarly, timber growth and harvesting patterns should be linked with rotation periods, and plantation in landscape patterns to reduce shifting and fire of forest tree species under climate-smart conditions for forest management to increase rural families' income for a sustainable agricultural ecosystem (Scherr et al., 2012). Although, conventional mitigation methods for the agriculture sector have a pivotal role in forest related strategies, some important measures are also included in which afforestation and reforestation should be increased but degradation and deforestation should be reduced and carbon sequestration can be increased (Spittlehouse, 2005; Seddon et al., 2018; Arehart et al., 2021). Carbon stock enhanced the carbon density of forest and wood products through longer rotation lengths and sustainable forest management (Rana et al., 2017; Sangareswari et al., 2018). Climate change impacts are reduced through adaptation strategies in agroforestry including tree cover outside the forests, increasing forest carbon stocks, conserving biodiversity, and reducing risks by maintaining soil health sustainability (Mbow et al., 2014; Dubey et al., 2019). Similarly, climate-smart soil management practices like reduction in grazing intensity, rotation-wise grazing, the inclusion of cover and legumes crops, agroforestry and conservation tillage, and organic amendments should also be promoted to enhance the carbon and nitrogen stocks in soil (Lal, 2007; Pineiro et al., 2010; Xiong et al., 2016; Garcia-Franco et al., 2018).

### Opportunities for fisheries and aquaculture

Sustainable economic productivity of fisheries and aquaculture requires the adaptation of specific strategies, which leads to minimizing the risks at a small scale (Hanich et al., 2018). Therefore, to build up the adaptive capacity of poor rural farmers, measures should be carried out by identifying those areas where local production gets a positive response from variations in climatic conditions (Dagar and Minhas, 2016; Karmakar et al., 2018). Meanwhile, the need to build the climate-smart capacity of rural populations and other regions to mitigate the harmful impacts of climate change should be recognized. In areas which have flooded conditions and surplus water, the integration of aquaculture with agriculture in these areas provides greater advantages to saline soils through newly adapted aquaculture strategies, i.e, agroforestry (Ahmed et al., 2014; Dagar and Yadav, 2017; Suryadi, 2020). To enhance the food security and living standards of poor rural families, aquaculture and artificial stocking engage the water storage and irrigation structure (Prein, 2002; Ogello et al., 2013). In Asia, rice productivity is increased by providing nutrients by adapting rice-fish culture in which fish concertedly consume the rice stem borer (Poonam et al., 2019). Food productivity can be enhanced by the integration of pond fish culture with crop-livestock systems because it includes the utilization of residues from different systems (Prein, 2002; Ahmed et al., 2014; Dagar and Yadav, 2017; Garlock et al., 2022). It is important to compete with future challenges in the system by developing new strains which withstand high levels of salinity and poorer quality of water (Kataria and Verma, 2018; Lam et al., 2019).

## Conclusion

Globally, and particularly in developing nations, variability in climatic patterns due to increased anthropogenic activity has become clear. Asia may face many problems because of changing climate, particularly in South Asian countries due to greater population, geographical location, and undeveloped technologies. The increased seasonal temperature would affect agricultural productivity adversely. Crop growth models with the assistance of climatic and economic models are helpful tools to predict climate change impacts and to formulate adaptation strategies. To respond to the adverse effects of climate change, sustainable productivity under climate-smart and resilient agriculture would be achieved by developing adaptation and mitigation strategies. AgMIP-Pakistan is a good specimen of climate-smart agriculture that would ensure crop productivity in changing climate. It is a multi-disciplinary plan of study for climate change impact assessment and development of the site and crop-specific adaptation technology to ensure food security. Adaptation technology, by modifications in crop management like sowing time and density, and nitrogen and irrigation application has the potential to enhance the overall productivity and profitability under climate change scenarios. The adaptive technology of the rice-wheat cropping system can be implemented in other regions in Asia with similar environmental conditions for sustainable crop production to ensure food security. Early warning systems and trans-disciplinary research across countries are needed to alleviate the harmful effects of climate change in vulnerable regions of Asia. Opportunities as discussed have the potential to minimize the negative effect of climate variability and change. This may include the promotion of agroforestry and mixed livestock and cropping systems, climate-smart water, soil, and energy-related technologies, climate resilient breeds for crops and livestock, and carbon sequestration to help enhance production under climate change. Similarly, the application of ICT-based technologies, EWS, AWS, and decision support systems for decision-making, precision water and nutrient management technologies, and crop insurance may be helpful for sustainable production and food security under climate change.

## Author contributions

AA, MH-u-R, and AR: conceptualization, validation, and formal analysis. MH-u-R, SAh, AB, WN, AE, HA, KH, AA, FM, YA, and MH: methodology, editing, supervision, and project administration. Initial draft was prepared by MH-u-R and improved and read by all co-authors. All authors contributed to the article and approved the submitted version.

## Funding

This research funded by the Deputyship for Research and Innovation, Ministry of Education in Saudi Arabia under grant number (IFPRP: 530-130-1442).

## Conflict of interest

The authors declare that the research was conducted in the absence of any commercial or financial relationships that could be construed as a potential conflict of interest.

## Publisher's note

All claims expressed in this article are solely those of the authors and do not necessarily represent those of their affiliated organizations, or those of the publisher, the editors and the reviewers. Any product that may be evaluated in this article, or claim that may be made by its manufacturer, is not guaranteed or endorsed by the publisher.
